# An Environmental Engineering Study Case: Constructing Cataluminescence Sensors Based on Octahedral Nanocomposites for Isovaleraldehyde Detection

**DOI:** 10.3390/molecules30030646

**Published:** 2025-02-01

**Authors:** Bai Sun, Mao Cai, Guoji Shi, Yun Wang, Lining Bao, Qiang Zhao, Mingjian Yi, Shuguang Zhu

**Affiliations:** 1 Anhui Institute of Urban and Rural Green Development and Urban Renewal, College of Environment and Energy Engineering, Anhui Jianzhu University, Hefei 230601, China; cm20233080@stu.ahjzu.edu.cn (M.C.); shi13215555415@163.com (G.S.); wangyun7943@163.com (Y.W.); baolining@ahjzu.edu.cn (L.B.); rommel99@163.com (Q.Z.); mjyi@ustc.edu.cn (M.Y.); zhushuguang@ahjzu.edu.cn (S.Z.); 2 Environmental Materials and Pollution Control Laboratory, Hefei Institute of Physical Science, Chinese Academy of Sciences, Hefei 230031, China

**Keywords:** cataluminescence, isovaleraldehyde, sensor, NiCo_2_O_4_/MIL-Fe_53_

## Abstract

Isovaleraldehyde is an important chemical raw material for the production of flavors, which is volatile and flammable and poses a health risk to humans. It is, therefore, essential to develop a rapid assay for the identification of isovaleraldehyde. In this study, octahedral NiCo_2_O_4_/MIL-Fe_53_ nanocomposites were successfully fabricated for the rapid detection of isovaleraldehyde. The prepared NiCo_2_O_4_/MIL-Fe_53_ nanocomposites were characterized by SEM, XRD, FTIR, and XPS to analyze the material properties. The effects of temperature, carrier gas flow rate, selectivity, and stability on the cataluminescence performance of this sensor were investigated. The results showed that NiCo_2_O_4_/MIL-Fe_53_ nanocomposites have excellent selectivity to isovaleraldehyde with response and recovery times of 6 and 8 s, respectively. A linear relationship was found between the CTL signal and isovaleraldehyde concentration Y = 9.56X − 23.3 (R^2^ = 0.99) over the concentration range of 13.66 to 437.22 ppm with a detection limit of 2.44 ppm. The relative deviation RSD = 4.18% for multiple tests of the sensor indicates good stability and longevity. Mechanistic studies have shown that the heterojunction formed by NiCo_2_O_4_/MIL-Fe_53_ nanocomposites has the advantage of improving CTL sensing performance. This study may advance the application of cataluminescence sensors in the detection of isovaleraldehyde.

## 1. Introduction

Volatile organic compounds (VOC) constitute a class of organic compounds that readily evaporate at an ambient temperature and pressure, and they are significant contributors to air pollution, posing detrimental effects on both human health and the environment and, in recent years, their detection has received widespread attention [1,2]. Among them, isovaleraldehyde is mainly used as an industrial raw material for the production of flavors, fragrances, and other organic chemicals [3], and it is important to note that vapor may form explosive mixtures with air. This means that exposure to open flames or high heat may cause combustion or an explosion. In high concentrations, it shows an irritating odor, which is irritating to the skin and respiratory system. Prolonged exposure to isovaleraldehyde vapor may cause dizziness, headache, nausea, and vomiting [4]. Developing a highly sensitive, simple, and rapid method to detect isovaleraldehyde is therefore important.

The contemporary methods with the capability to detect isovaleraldehyde gas include high-performance liquid chromatography (HPLC), gas chromatography (GC), and gas chromatography–mass spectrometry (GC-MS), amongst others. Quantitative analysis of isovaleraldehyde in Japanese sake was reported by using high-performance liquid chromatography by Masayuki Takahashi et al. [5]. GC-MS was used to separate and characterize the volatile compounds in shochu by Yukio Osafune et al. [6]. While these methodologies offer accurate detection of isovaleraldehyde, they are not without their limitations, such as the large size of the detection instruments, cumbersome procedures, and prolonged detection times, which are detrimental to rapid detection [7,8]. In 1976, Breysse et al. discovered a weak luminescence during the catalytic oxidation of CO on the surface of ThO_2_ [9]. This phenomenon was named cataluminescence (CTL). Subsequently, in the 1990s, Nakagawa et al. reported a CTL sensor for the detection of alcohols and ketones using γ-Al_2_O_3_ as catalytic material [10,11]. The principle of the CTL sensor is that the CTL phenomenon occurs when the substance to be measured comes into contact with the CTL material, and the target substance is detected by the light signal generated by the CTL reaction [12,13]. In recent years, CTL sensors have been widely developed and applied in the field of VOC monitoring due to their advantages of low cost, compact and convenient use, high sensitivity, and good stability [14,15]. Construction of a CTL sensor system for acetone vapor detection was conducted by Shi G et al. [16]. An In_2_S_3_ CTL sensor was developed for the detection of ammonium sulfide by Cai P et al. [17].

Metal–organic frameworks (MOFs), which are porous structural compounds assembled through the coordination of metal ions or clusters with organic ligands, exhibit substantial potential across various domains due to their distinctive structural attributes [18,19]. Due to their well-defined geometry, large specific surface area, and stability [20,21], MOF materials are widely used in the fields of absorption [22], photocatalysis [23,24], and advanced oxidation [25]. With the deeper exploration of sensor research, MOF materials are gradually being explored and applied to the field of CTL [26,27]. Huang X et al. added UIO-66 material to a CTL sensor to detect acetone vapor, resulting in a 25-fold enhancement of the CTL signal [28]. Li Q et al. synthesized a Ce(IV)-MOF and utilized it for the development of a highly sensitive H_2_S gas sensor, which shows promising applications in H_2_S detection [29]. The incorporation of MOF materials in the development process of CTL sensors has significantly enhanced the CTL performance of the sensors.

MIL-Fe_53_(C_8_H_4_FeO_5_) is a class of MOFs synthesized from iron(III) cations and 1,4-benzenedicarboxylate, characterized by a three-dimensional network or secondary building units comprising hexagonal FeO_6_ chains and dicarboxylate anions [30]. MIL-Fe_53_ is chemically stable and is made up of less toxic metal centers compared to other MOFs and has, therefore, received a great deal of attention [30]. Sun Y H et al. have successfully fabricated an electrochemical sensor based on MIL-Fe_53_, demonstrating its efficacy in the detection of fluosilazole, thereby highlighting its potential applications in the sensing of this specific compound [31]. NiCo_2_O_4_ is a p-type oxide semiconductor that exhibits several advantageous properties, including its low cost, environmental friendliness, structural stability, and abundant electroactive sites. Consequently, NiCo_2_O_4_ is used for the monitoring of volatile organic compounds. Wu J et al. have fabricated a gas sensor based on NiCo_2_O_4_ nanocomposite material, which exhibits efficacy in the detection of hydrogen sulfide [32]. However, NiCo_2_O_4_, as a p-type oxide semiconductor, has a response rate that is generally lower compared to the majority of n-type semiconductors. In pursuit of enhanced performance for CTL sensors, it is imperative to identify methods capable of optimizing their sensing characteristics. A substantial specific surface area and a stable structure are advantageous traits in this regard. Kim S H et al. combined NiCo_2_O_4_ and Pd to improve the sensitivity of the sensor against H_2_ [33]. Ghanbarian M et al. developed MIL-53 (Cr-Fe)/Ag/CNT ternary nanocomposites for the preparation of gas sensors [34].

Therefore, this study is aimed at combining the advantages of NiCo_2_O_4_ and MIL-Fe_53_ to improve CTL’s performance. The successful synthesis of NiCo_2_O_4_/MIL-Fe_53_ nanocomposites was achieved for the purpose of preparing a CTL sensor for the detection of isovaleraldehyde gas. A comprehensive study of the effects of reaction temperature, carrier gas flow rate, selectivity, and stability on CTL performance and sensor sensing characteristics was carried out. The potential CTL mechanism is also discussed.

## 2. Results and Discussion

### 2.1. Characterization of Material

**Figure 1:**
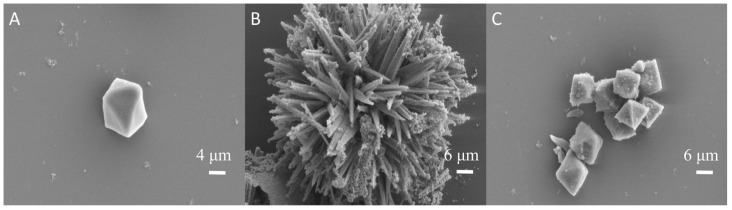
(**A**) The SEM images of MIL-Fe_53_, (**B**) NiCo_2_O_4_, and (**C**) NiCo_2_O_4_/MIL-Fe_53_.

The morphology of the synthesized materials was studied on a scanning electron microscope (SEM). Figure 1A–C shows the SEM images of MIL-Fe_53_, NiCo_2_O_4_, and the nanocomposites, respectively. As depicted in Figure 1A, it can be observed that MIL-Fe_53_ has an octahedral bipyramidal morphology with an average diameter of 12 µm and a uniform distribution, and such a smooth surface endows MIL-Fe_53_ with a larger specific surface area. As depicted in Figure 1B, it is observable that NiCo_2_O_4_ consists of external nanoparticles forming a sea urchin-like structure, which is in line with descriptions of the spinel structure in the literature, indicating that the structure of NiCo_2_O_4_ is not damaged during the calcination process. Figure 1C shows that the nanocomposites consist of octahedral MIL-Fe_53_ and NiCo_2_O_4_ particles attached to their surface, and the organometallic skeleton of the MOF is not disrupted after the material is composited. The regular octahedral morphology and smooth surface of MIL-Fe_53_ provide more attachment sites for NiCo_2_O_4_ and also facilitate the formation of heterostructures between MOF materials and metal oxides. The NiCo_2_O_4_, which is integrated onto the surface of MIL-Fe_53_ octahedra, forms a robust nanocomposite material that enhances the stability of the material. Concurrently, the surface roughness of the material is increased, providing favorable conditions for the adsorption of target substances during the reaction process.

**Figure 2:**
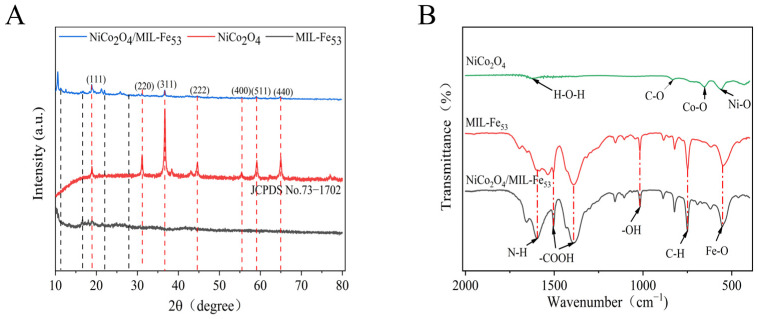
(**A**) XRD patterns of NiCo_2_O_4_/MIL-Fe_53_ nanocomposites; (**B**) FTIR spectra of NiCo_2_O_4_, MIL-Fe_53_, and NiCo_2_O_4_/MIL-Fe_53_.

The composition and crystal structure of the nanocomposite material were determined by using X-ray diffraction (XRD), with the experimental results depicted in Figure 2. Figure 2A shows the XRD patterns of NiCo_2_O_4_, MIL-Fe_53_, and NiCo_2_O_4_/MIL-Fe_53_ nanocomposites. The diffraction peaks at 2θ values of 18.93°, 31.15°, 36.7°, 44.63°, 55.73°, 59.11°, and 64.96° correspond to the (111), (220), (311), (222), (400), (511), and (440) crystal planes of NiCo_2_O_4_ in the nanocomposite material, respectively, which are in agreement with the JCPDS No. 73−1702 card. For MIL-Fe_53_ in nanocomposites, XRD shows diffraction peaks at 2θ = 11.1°, 16.3°, 17.7°, 18.1°, and 22.3°, which is in agreement with the MIL-Fe_53_ reported in the literature [35]. It can be observed from Figure 2A that there are obvious diffraction peaks of the NiCo_2_O_4_/MIL-Fe_53_ nanocomposites. Figure 2B demonstrates the Fourier transform infrared spectrometer (FTIR) spectra of the single material and the NiCo_2_O_4_/MIL-Fe_53_ nanocomposite material. The characteristic peak at position 1655 cm^−1^ corresponds to the bending vibration of the N-H group in the amide feedstock. The presence of a dicarboxylic acid linker in MIL-Fe_53_ was indicated by the detection of typical symmetric and asymmetric vibrations of -COOH at peaks of 1385 cm^−1^ and 1535 cm^−1^ [36], respectively. The characteristic peaks observed at 1010 cm^−1^ and 748 cm^−1^ are attributed to the bending vibrations of the hydroxyl group -OH and the benzene ring C-H in the organic linkers [37], respectively. Indeed, the absorption peak situated at 538 cm^−1^ is indicative of the stretching vibration of the Fe-O bond, which is a distinctive functional group within the structure of MIL-Fe_53_. This vibrational mode is characteristic of the metal–oxygen interaction within the framework [38]. The absorption peaks at the low wavenumbers of 552 cm^−1^ and 650 cm^−1^ are attributed to the stretching vibrations of Ni-O and Co-O in NiCo_2_O_4_, which are indicative of the metal–oxygen bonds within the spinel structure of NiCo_2_O_4_ [39]. NiCo_2_O_4_ exhibits a C-O stretching vibration absorption peak within the range of 1000 cm^−1^ to 650 cm^−1^, which can be associated with the vibrational modes of the carbon–oxygen bonds in the structure. Additionally, an absorption peak at 1633 cm^−1^ is observed, which can be attributed to the bending vibration of water molecules in the interlayer of Ni(OH)_2_. This peak is likely a result of the interaction between NiCl_2_·6H_2_O and water molecules during the preparation of NiCo_2_O_4_, where part of the NiCl_2_·6H_2_O binds with water molecules, leading to the formation of this characteristic peak.

**Figure 3:**
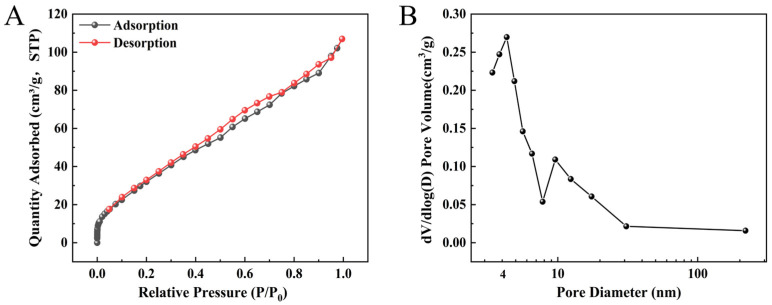
(**A**) Adsorption isotherms of NiCo_2_O_4_/MIL-Fe_53_ nanocomposites; (**B**) pore size analysis plots of NiCo_2_O_4_/MIL-Fe_53_ nanocomposites.

In order to investigate the porosity of NiCo_2_O_4_/MIL-Fe_53_ nanocomposites, the adsorption-desorption of N_2_ was used to obtain the adsorption and desorption isotherms. The specific surface area and pore size distribution of the samples were calculated according to the Barret-Joyner-Halenda (BJH) model, and the specific surface area of NiCo_2_O_4_/MIL-Fe_53_ nanocomposites was 138.246 m^2^/g. As can be seen in Figure 3A, the nanocomposites exhibit type-II isotherm characteristics, reflecting the physical adsorption process of porous adsorbents. The ability of rapid physisorption of N_2_ when the relative pressure is in the range of 0 to 0.01 proves that a large number of pores exist in the nanocomposites, and the N_2_ adsorption at higher relative pressures is related to the presence of mesopores within the nanocomposites, and the rapid rise is mainly due to the formation of mesopores between the particles. Combined with Figure 3B, it can be seen that the pore sizes of the nanocomposites are mainly distributed in the range of 1–10 nm, probably due to the accumulation between NiCo_2_O_4_/MIL-Fe_53_ particles, which ultimately forms a mesoporous structure.

**Figure 4:**
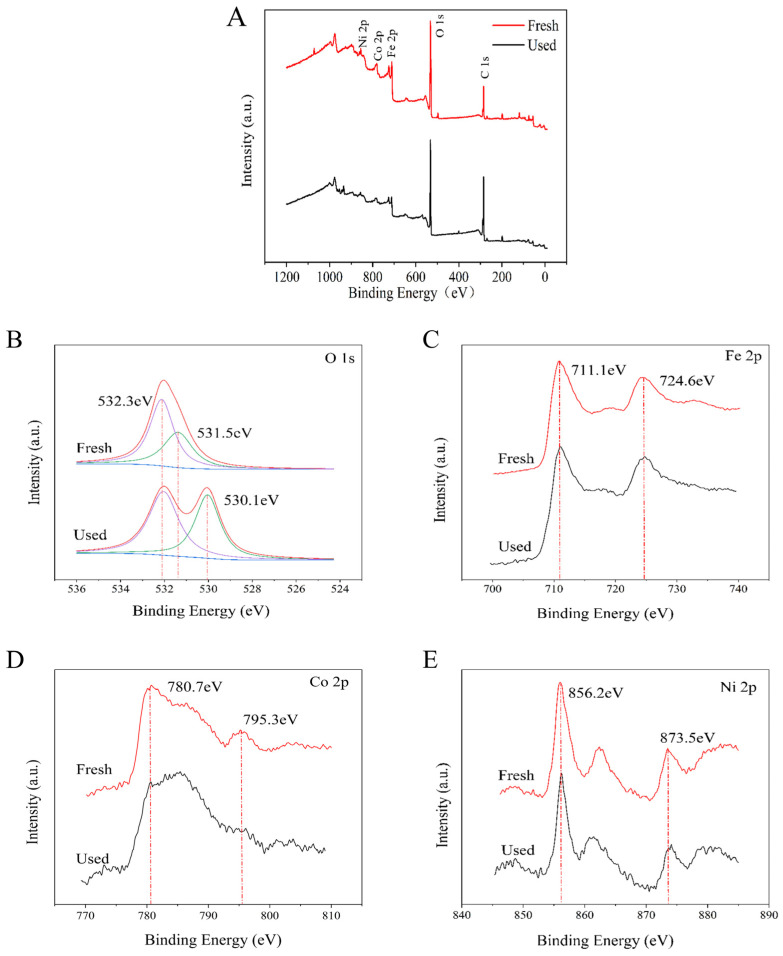
(**A**) XPS curves of fresh and used reaction NiCo_2_O_4_/MIL-Fe_53_; (**B**) high-resolution scan of O 1 s; (**C**) high-resolution scan of Fe 2p; (**D**) high-resolution scan of Co 2p; and (**E**) high-resolution scan of Ni 2p.

The elemental composition and changes of NiCo_2_O_4_/MIL-Fe_53_ nanocomposites before and after the reaction were analyzed by X-ray photoelectron spectroscopy (XPS) characterization. Figure 4A presents the full XPS spectra of the nanocomposite material before and after the reaction. It can be observed that the elements before and after the reaction still correspond to their respective orbitals, indicating the presence of elements Fe, Co, Ni, and O in the nanocomposite material, as well as C, which is adsorbed from the carrier gas. The XPS spectrum of O1s in Figure 4B shows that the oxygen in the material is mainly dominated by the lattice oxygen (O_L_) at 532.2 eV and the oxygen vacancy (O_V_) at 531.5 eV before the reaction. After the reaction, it can be clearly seen that the peak position of O_L_ remains unchanged, but the peak area increases and the O_V_ is shifted to the right from 531.5 to 530.1 eV, with an increasing peak area. This is because before the start of the reaction, the carrier gas was just passed into the reaction chamber due to the heating rod temperature not reaching the appropriate reaction temperature, resulting in the reaction of dissociated oxygen content being relatively small, the number of oxygen vacancies is insufficient, and the O_L_ content obtained by reduction is naturally less; when the temperature rises to the appropriate reaction temperature of the material, the dissociation of oxygen accelerates, the content of oxygen vacancies rises, and the whole process of the redox reaction is accelerated, and the content of the O_L_ also rises accordingly. The results of the XPS show that the accelerated formation of oxygen species can accelerate the efficiency of the CTL reaction from the inside. The XPS fine maps of Fe 2p, Co 2p, and Ni 2p before and after the reaction are shown in Figure 4C, Figure 4D, and Figure 4E, respectively, from which it can be seen that the positions of the characteristic peaks before and after the reaction are basically unchanged, indicating that there is no major change in the materials before and after the reaction, which reflects the effect of the nanocomposites as catalysts in the CTL reaction.

### 2.2. CTL Performance Study of NiCo_2_O_4_/MIL-Fe_53_

**Figure 5:**
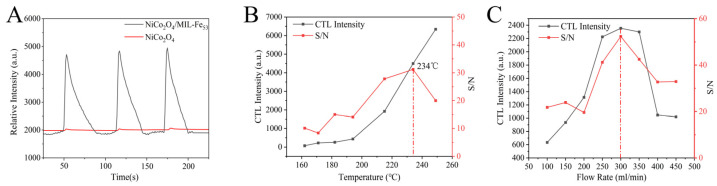
(**A**) CTL signal intensities of NiCo_2_O_4_, NiCo_2_O_4_/MIL-Fe_53_ against isovaleraldehyde; (**B**) effect of working temperature on CTL signal intensity and S/N (concentration: 156.16 ppm; flow rate: 350 mL/min); (**C**) effect of carrier gas flow rate on CTL signal intensity and S/N (concentration: 156.16 ppm; temperature: 220 °C).

The influence of the incorporation of MIL-Fe_53_ on the CTL signal of NiCo_2_O_4_ has been investigated. Figure 5A shows the response signal intensities for CTL of isovaleraldehyde by a single NiCo_2_O_4_ material and NiCo_2_O_4_/MIL-Fe_53_ nanocomposites; the red line is the signal of a single NiCo_2_O_4_ material, and the black line is the signal of the NiCo_2_O_4_/MIL-Fe_53_ nanocomposite material. It is evident from the figure that the CTL signal strength of the nanocomposite sensor is very significantly improved compared to NiCo_2_O_4_, which indicates that the MIL-Fe_53_ component in the nanocomposite material really improves the performance of the sensor. In order to be able to better study the CTL properties of NiCo_2_O_4_/MIL-Fe_53_ nanocomposite sensors, the effects of the reaction temperature and carrier gas flow rate on the CTL performance of the NiCo_2_O_4_/MIL-Fe_53_ nanocomposite sensors have been explored.

Figure 5B shows the CTL signal intensity and signal-to-noise ratio of NiCo_2_O_4_/MIL-Fe_53_ nanocomposites for isovaleraldehyde at different temperatures. The temperature range tested in the experiment was from 162 °C to 250 °C. Within the temperature range from 162 °C to 250 °C, the overall CTL signal, represented by the black line, increases with the rising temperature. Specifically, the signal increases gradually within the range from 162 °C to 194 °C and more rapidly within the range from 194 °C to 250 °C. The signal-to-noise ratio (S/N) of the red line increased with the temperature within the range of 162 °C to 234 °C, decreased with the temperature within the range of 234 °C to 250 °C, and reached a maximum value of 31 at 234 °C. Combining the CTL signal and the S/N ratio, it can be seen that the peak intensity of the CTL signal and the signal-to-noise ratio of the nanocomposites reach relatively high values at 234 °C. Therefore, 234 °C was selected as the optimum control temperature.

Figure 5C presents the CTL signal intensity and S/N of the NiCo_2_O_4_/MIL-Fe_53_ nanocomposites under various carrier gas flow rates. The range of carrier gas flow rates tested in the experiment was from 100 mL/min to 450 mL/min. It was observed that the CTL signal and S/N ratio increased with the increase in the carrier gas flow rate when it was between 100 mL/min and 300 mL/min. This enhancement may be attributed to the fact that a slower flow rate could lead to the dilution of isovaleraldehyde concentration upon entering the reaction chamber, consequently resulting in a lower CTL signal. Within the range of 300 mL/min to 450 mL/min, the CTL signal and S/N decreased with the increase in carrier gas flow rate. This decrease is attributed to the rapid flow rate leading to a shortened contact time between isovaleraldehyde and the sensor upon entry into the reaction chamber, thereby affecting the signal intensity. At a carrier gas flow rate of 300 mL/min, both the CTL intensity and S/N ratio reached their maxima, which is why 300 mL/min was selected as the optimal control flow rate.

**Figure 6:**
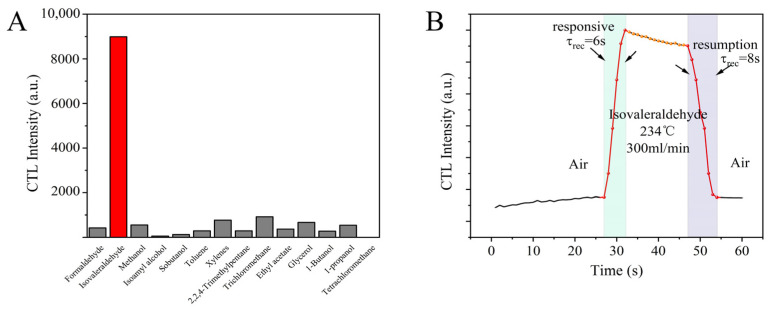
(**A**) CTL response of the sensor to different compounds (temperature: 234 °C; flow rate: 300mL/min); (**B**) characterization of the response of the isovaleraldehyde sensor.

Based on the aforementioned studies, the optimal laboratory control conditions of 234 °C and 300 mL/min were established, and under these conditions, the CTL characteristics of the NiCo_2_O_4_/MIL-Fe_53_ nanocomposites-based CTL sensor in the analysis of isovaleraldehyde were investigated. Figure 6A shows the intensity of the CTL signals of the NiCo_2_O_4_/MIL-Fe_53_ nanocomposite sensor for different volatile organic compounds under optimum control conditions. It can be seen that the NiCo_2_O_4_/MIL-Fe_53_ nanocomposite sensor has the highest CTL signal for isovaleraldehyde and basically no signal for other organics, such as toluene and formaldehyde, except for trichloromethane and xylenes, which have a weak signal response. This proves that the NiCo_2_O_4_/MIL-Fe_53_ nanocomposite prepared in this experiment has high selectivity to isovaleraldehyde and is an excellent CTL sensing material for isovaleraldehyde. Figure 6B shows the CTL response characteristics of the NiCo_2_O_4_/MIL-Fe_53_ nanocomposite sensor to isovaleraldehyde under optimal control conditions. Upon the introduction of isovaleraldehyde into the reaction chamber facilitated by the carrier gas, the NiCo_2_O_4_/MIL-Fe_53_ nanocomposite sensor responded rapidly, and the CTL signal increased to reach the maximum value with a response time of 6 s, and when the isovaleraldehyde was completely reacted, the CTL signal began to return to the initial state, with a recovery time of 8 s. The response and recovery time of the sensor for CTL against isovaleraldehyde is excellent, which reflects the advantages of the fast response of the CTL sensor.

**Figure 7:**
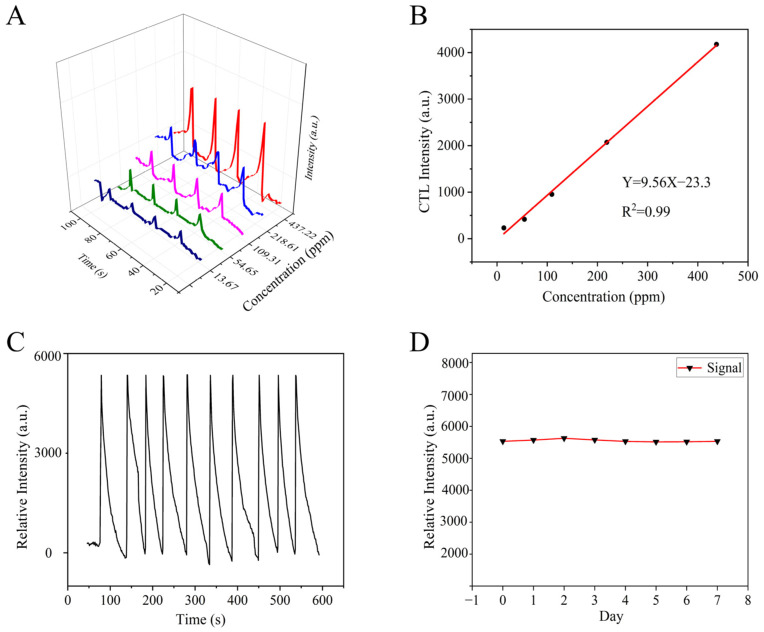
(**A**) CTL intensities corresponding to different concentrations of isovaleraldehyde; (**B**) curve of isovaleraldehyde concentration versus signal intensity; (**C**) signal intensity of 10 tests of NiCo_2_O_4_/MIL-Fe_53_ sensor over 600s; and (**D**) signal intensity of NiCo_2_O_4_/MIL-Fe_53_ sensor tested over seven days.

In order to elucidate the relationship between the catalytic signals of the NiCo_2_O_4_/MIL-Fe_53_ nanocomposite sensor and the concentration of the analyte, a systematic investigation is warranted. Isovaleraldehyde gas was injected through different concentrations (13.66, 54.65, 109.31, 218.61, and 437.22 ppm) under optimal control conditions. As depicted in Figure 7A, distinct colored lines represent the CTL signals corresponding to varying concentrations of isopentanal. It is observed that with the increment of isopentanal concentration, there is a marked escalation in the CTL intensity. Figure 7B illustrates the fitted linear relationship between the isovaleraldehyde concentration and CTL signal intensity; the fitted equation in the concentration range of 13.66–437.22 ppm is Y = 9.56X − 23.3 (R^2^ = 0.99), from which it can be seen that the CTL signal of the sensor has a good and positive correlation to the concentration of isovaleraldehyde. The detection limit of the isovaleraldehyde concentration was calculated using Equation (1).

(1) $$D=\frac{3N\times Q}{I}$$

In this context, *Q* denotes the minimum analyte concentration within the detection range; *N* signifies the response noise at the minimum analyte concentration; and *I* corresponds to the response signal value associated with the minimum analyte concentration within the detection range. The calculated detection limit is determined to be 2.44 ppm. In addition, the NiCo_2_O_4_/MIL-Fe_53_ sensor is compared to some reports. It is noted that since there is rarely a report about isovaleraldehyde gas sensors, the reported sensors for detecting aldehyde (hexanal) gas, which is similar to isovaleraldehyde, are employed for comparison. For example, Janfaza et al. reported a sensor based on MIP-MWCNTs, which showed a response/recovery time of 300/200 s with a detection limit of 10 ppm [40]. Yao et al. prepared a MnO_2_/Ti_3_C_2_Tx-based sensor for detecting aldehyde, which exhibited a response/recovery time of 138/318 s with a detection limit of 20 ppm [41]. It can be seen that the NiCo_2_O_4_/MIL-Fe_53_ sensor presented here displays high performances, including short response/recovery times and a low detecting limit.

Furthermore, the stability of the CTL signal is an essential parameter for the evaluation of sensor performance. From Figure 7C, it can be seen that the CTL signals obtained from 10 consecutive tests in 600 s time are all around 5500, and the results show that the CTL signal strength of the isovaleraldehyde sensor is relatively stable, indicating that the NiCo_2_O_4_/MIL-Fe_53_ nanocomposite has good stability. For seven days after the NiCo_2_O_4_/MIL-Fe_53_ nanocomposite sensors were prepared, the sensor signal levels to isovaleraldehyde were repeatedly tested each day under the same control conditions. Figure 7D shows the CTL signal intensity under the best working conditions in one week. It can be seen that the fluctuation of the sensor signal intensity measured in 7 days is small, the reproducibility is good, and the relative deviation (RSD = 4.18%) of repeated tests under the same conditions over a period of 7 days is less than 5%. These data indicate that the catalytic sensor maintains good stability and service life under continuous operating conditions, which meets the practical application requirements for sensors.

### 2.3. Possible CTL Mechanisms

**Figure 8:**
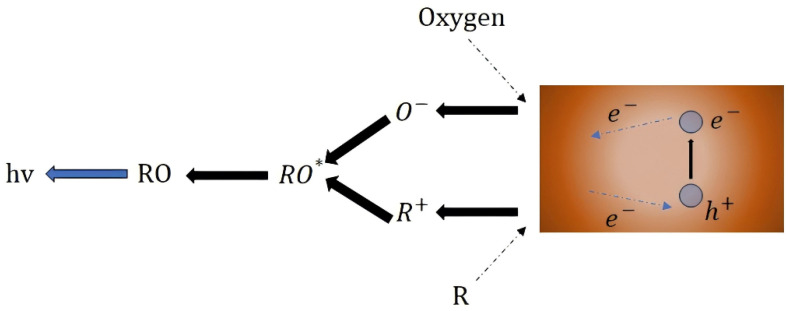
Reaction mechanism diagram of cataluminescence.

It is considered that the combination of MIL-Fe_53_ and NiCo_2_O_4_ enhances the conductivity and improves its electronic structure. The molecular activity of NiCo_2_O_4_/MIL-Fe_53_ is activated when a certain temperature threshold is reached on the surface of the ceramic heating line. Isovaleraldehyde enters the reaction chamber through the carrier gas and contacts the CTL sensor. Oxygen in the carrier gas will be firstly adsorbed on the surface of the nanocomposite to form O_2_ (ads) in the adsorbed state (Equation (2)), and then O_2_ will gain electrons from the conduction band of the NiCo_2_O_4_/MIL-Fe_53_ nanocomposite to become O_2_^−^ (Equation (3)) in the adsorbed state and O^−^ (Equation (4)) [42]. An electron depletion layer will be formed on the surface of the material, and the nanocomposite exhibits a highly active state. The MIL-Fe_53_ provides attachment sites for NiCo_2_O_4_ to enable more channels for the transfer of free electrons and accelerate the catalytic efficiency. Then comes the oxidation stage of isovaleraldehyde, where the first three reactions are more and more intense, the activated NiCo_2_O_4_/MIL-Fe_53_ nanocomposite material is in contact with isovaleraldehyde molecules, and the chemisorbed O^−^ on the surface of the material will undergo oxidation with isovaleraldehyde molecules to generate CO_2_ and H_2_O (Equation (5)). The CO_2_ generated by the reaction is the excited state intermediate carbon dioxide (CO_2_*), which is accompanied by the return of the intermediate to the ground state (Equation (6)), releasing the light signal hν [43]. This weak light will be detected by the BPCL instrument and ultimately converted into a signal received by the sensor. The flowchart of the whole reaction process is shown in Figure 8.

(2) $$O_{2}(\mathrm{gas})\to O_{2}(\mathrm{ads})$$

(3) $$O_{2}(\mathrm{ads})+e^{-}\to O_{2}^{-}(\mathrm{ads})$$

(4) $$O_{2}^{-}(ads)+e^{-}\to 2O^{-}(ads)$$

(5) $$C_{5}H_{10}O+7O^{-}\to 5CO_{2}^{*}+5H_{2}O+{7e}^{-}$$

(6) $$CO_{2}^{*}\to CO_{2}+h\nu$$

**Figure 9:**
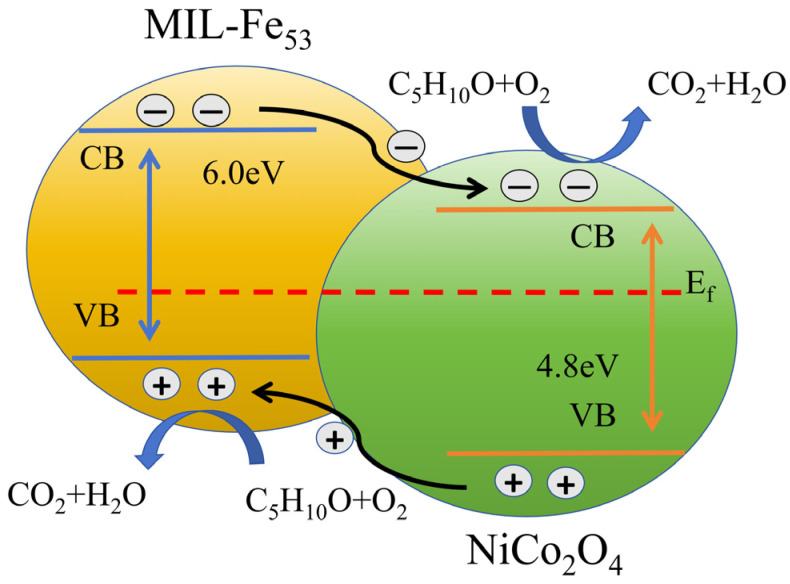
Mechanism of heterojunctions in NiCo_2_O_4_/MIL-Fe_53_ nanocomposites.

During the CTL reaction, NiCo_2_O_4_ grows on the surface of MIL-Fe_53_ to form a heterojunction, and this heterojunction can achieve spatial separation of electrons and holes on the surface of the sensing material, which generates strongly oxidizing and reducing holes and electrons on the surface of the heterojunction and participates in the catalytic reaction of isovalerylaldehyde, which has a better catalytic effect than that of the ordinary free electrons. Although the construction of heterojunctions hinders the electron–hole nanocomposites to some extent, the material is prompted to form more electron–hole pairs [44,45], which improves the conversion efficiency of carriers and accelerates the reaction process. The forbidden bands (Eg) of MIL-Fe_53_ and NiCo_2_O_4_ were obtained by UV spectrophotometry and DFT theoretical calculations as 6.0 eV and 4.8 eV [46]. Based on the CTL response characteristics of isovaleraldehyde with the NiCo_2_O_4_/MIL-Fe_53_ nanocomposites, the CTL response mechanism of NiCo_2_O_4_/MIL-Fe_53_ to isovaleraldehyde is inferred, as shown in Figure 9. After isovaleraldehyde enters the reaction chamber through the carrier gas, the nanostructures on the surface of the nanocomposites adsorb isovaleraldehyde on the surface of the ceramic heating rod and, at the same time, excite the generated electrons (*e*^−^) to be transferred from the MIL-Fe_53_ conduction band (CB) to the NiCo_2_O_4_ CB [47], and the generated holes (*h^+^*) on the NiCo_2_O_4_ valence band (VB) will jump to the MIL-Fe_53_ VB, so that *e^−^* and *h^+^* are located on NiCo_2_O_4_ and MIL-Fe_53_, respectively, to realize the effective separation of *e^−^* and *h^+^.* When MIL-Fe_53_ is added, the Fermi energy level difference (*E_fn_ − E_fp_*) [48,49] between NiCo_2_O_4_ and MIL-Fe_53_ increases, and more strong oxidizing *h^+^* and strong reducing *e*^−^ will be generated on the surface of the heterojunction formed by NiCo_2_O_4_ and MIL-Fe_53_ [50,51], which directly or indirectly participates in the CTL reaction of isovaleraldehyde, and it can effectively improve the CTL ability of NiCo_2_O_4_/MIL-Fe_53_ nanocomposites.

## 3. Materials and Methods

### 3.1. Chemical Reagents

The chemical reagents used in this study include the following: ferric chloride hexahydrate (FeCl_3_·6H_2_O; 99.0%; Beijing Wokai Biotechnology Co., Beijing, China); N-methylacetamide (C_3_H_7_NO; 99.5%; Beijing Wokai Biotechnology Co., Beijing, China); terephthalic acid (C_8_H_6_O_4_; 99.0%; Beijing Wokai Biotechnology Co., Beijing, China); anhydrous methanol (CH_4_O; 99.5%; Xilong Science Co., Shantou, China); cobalt chloride hexahydrate (CoCl_2_·6H_2_O; 99.0%; Xilong Science Co., Shantou, China); nickel chloride hexahydrate (NiCl_2_·6H_2_O; 98.0%; Xilong Science Co., Shantou, China); urea CH_4_N_2_O; 99.0%; Beijing Wokai Biotechnology Co.); and anhydrous ethanol (C_2_H_6_O, 99.5%, Zhengzhou Yibang Industry Co., Zhengzhou, China).

### 3.2. Instrumentation

The analytical instruments used during this study are as follows: A BPCL weak luminescence meter (BPCL-1; Guangzhou Microluminescence Science and Technology Co., Guangzhou, China); X-ray diffractometer (X’pertPRO MPD. Panalytical; Almelo, The Netherlands); scanning electron microscope (AURIGA; ZEISS, Jena, Germany); Fourier transform infrared spectrometer (NEXUS-870; ThermoFisher, Waltham, MA, USA); and X-ray photoelectron spectrometer (ESCALAB 250; ThermoFisher, Waltham, MA, USA).

### 3.3. Synthesis of NiCo_2_O_4_/MIL-Fe_53_

NiCo_2_O_4_/MIL-Fe_53_ nanocomposites were synthesized by the hydrothermal method. In the first step, NiCo_2_O_4_ monomer material was synthesized first. A solution was prepared by dissolving 1 mmol of NiCl_2_·6H_2_O and 2 mmol of CoCl_2_·6H_2_O in a mixture of 20 mL of ultrapure water and 20 mL of ethanol and then adding 15 mmol of urea and stirring it until dissolved. It was then transferred to an autoclave and reacted for 12 h at 120 °C, with centrifugal washing and drying for 24 h at 60 °C. Afterward, crushing and annealing took place at 350 °C for 3 h to obtain a black NiCo_2_O_4_ powder. In the second step, the NiCo_2_O_4_ monomer material was compounded with MIL-Fe_53_. The prepared 1 mmol of NiCo_2_O_4_ was added to 110 mL of DMF and treated with ultrasonication. Then, 4 mmol of FeCl_3_·6H_2_O and 5 mmol of H_2_BDC were added, stirred until dissolved and loaded into an autoclave. Then, the reaction mixture was subjected to a thermal treatment at 150 °C for a duration of 12 h, followed by cooling to room temperature, and then centrifuged and washed and dried at 60 °C for 12 h, and finally, the NiCo_2_O_4_/MIL-Fe_53_ powder was obtained.

### 3.4. CTL Sensing Measurements

**Figure 10:**
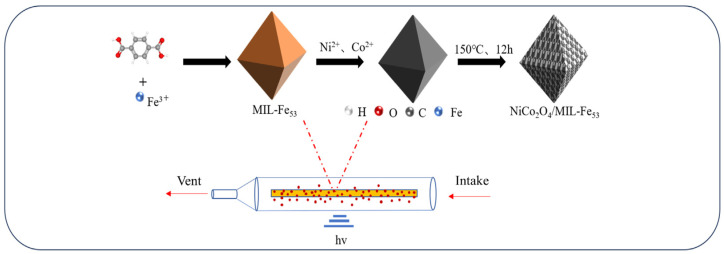
Diagram of the synthesized structure of NiCo_2_O_4_/MIL-Fe_53_ and schematic diagram of the BPCL sensing device.

The three-dimensional morphological architecture of the NiCo_2_O_4_/MIL-Fe_53_ nanocomposite throughout its synthesis and the schematic depiction of the Ultra-Weak Luminescence Analyzer (BPCL) detection setup are delineated in Figure 10. The study design involved attaching a needle-like NiCo_2_O_4_ to octahedral MIL-Fe_53_ to form a nanocomposite material and then attaching the nanocomposite material to a ceramic heating rod. Within the quartz reaction chamber, isovaleraldehyde molecules were transported by the carrier gas stream and came into contact with the NiCo_2_O_4_/MIL-Fe_53_ nanocomposite material. This interaction subsequently triggers a reaction and leads to the emission of CTL. Finally, the CTL signal hν was detected by the BPCL instrument and converted into data for analysis.

## 4. Conclusions

In this study, a CTL sensor based on an octahedral NiCo_2_O_4_/MIL-Fe_53_ nanocomposite was successfully developed for the rapid detection of isovaleraldehyde. The SEM, XRD, and FTIR et al. characterization results show that NiCo_2_O_4_/MIL-Fe_53_ nanocomposites were successfully synthesized, retaining the organometallic skeleton of a MOF. The influence of temperature and carrier gas flow rate on the CTL intensity of the nanocomposite sensor indicates that under the controlled conditions of 234 °C and 300 mL/min, the CTL signal intensity and S/N ratio are at their highest. Under optimal conditions, the nanocomposite sensors exhibited excellent selectivity and response characteristics to isovaleraldehyde, with a response time of 6 s and a recovery time of 8 s. Within the concentration range of 13.66 to 437.22 ppm, a good linear relationship was observed between the isovaleraldehyde concentration and CTL signal intensity, with the fitted linear equation of Y = 9.56X − 2.30 (R^2^ = 0.99) and a detection limit of 2.44 ppm. The relative deviation (RSD = 4.18%) of the sensor for multiple tests was less than 5%, which indicates that the sensor has good stability and service life. NiCo_2_O_4_/MIL-Fe_53_ nanocomposites showed significant CTL signal enhancement over single NiCo_2_O_4_. The mechanism was explored, and the results showed that the heterojunction formed by the addition of MIL-Fe_53_ and the nanocomposites of NiCo_2_O_4_ played an important role in the CTL reaction, and according to the XPS results, the increase in oxygen vacancies improved the efficiency of the CTL reaction. In conclusion, this study developed a NiCo_2_O_4_/MIL-Fe_53_ nanocomposite sensor for the rapid detection of trace isovaleraldehyde, which is expected to be useful in future environmental monitoring and industrial production. It also expanded the material exploration in the field of CTL, and it is expected to develop more CTL sensors for monitoring VOC through the nanocomposite composites of different materials in the future.

## Data Availability

Data are contained within the article.
